# Phytoremediation of Wastewater Containing Lead and Manganese Ions Using Algae

**DOI:** 10.3390/biology12060773

**Published:** 2023-05-26

**Authors:** Loredana Ioana Diaconu, Cristina Ileana Covaliu-Mierlă, Oana Păunescu, Leon Dumitru Covaliu, Horia Iovu, Gigel Paraschiv

**Affiliations:** 1 Faculty of Biotechnical Systems Engineering, University Politehnica of Bucharest, 313 Splaiul Independentei, 060042 Bucharest, Romania; 2 Faculty of Chemical Engineering and Biotechnologies, University Politehnica of Bucharest, 1–7 Gheorghe POLIZU Street, Sector 1, 011061 Bucharest, Romania

**Keywords:** phytoremediation, algae, heavy metal, removal, wastewater

## Abstract

**Simple Summary:**

The article provides important information about water treatment using algae. Waters are becoming increasingly polluted, and different methods are being studied that offer many advantages to treat them. In this context, the aim of this research is to share the results of experimental research carried out applying this phytoremediation process. The removal efficiencies of manganese and lead ions from wastewater are found to be 95.73% and 99.46% using *Sargassum fusiforme*, respectively, and 98.17% and 100.00% using *Enteromorpha prolifera*, respectively. Some advantages of applying this phytoremediation process for wastewater treatment are low cost, efficiency and plant sustainability. Thus, phytoremediation can be a very good approach for economical and sustainable remediation of waters containing organic or inorganic pollutants.

**Abstract:**

Heavy metal pollution of water from industrial discharge is a major problem worldwide. Thus, the quality of the environment and human health are severely affected. Various conventional technologies have been applied for water treatment, but these can be expensive, especially for industrial water treatment, and may have limited treatment efficiencies. Phytoremediation is a method that is successfully applied to remove metal ions from wastewater. In addition to the high efficiency of the depollution treatment, this method has the advantages of a low cost of the operation and the existence of many plants that can be used. This article presents the results of using algae (*Sargassum fusiforme* and *Enteromorpha prolifera*) to treat water containing manganese and lead ions. It was observed that maximum efficiencies for wastewater treatment were obtained when was used the algae *Enteromorpha prolifera* for a 600 min contact time period. The highest wastewater treatment efficiency obtained using *Sargassum fusiforme* was 99.46%.

## 1. Introduction

Despite the positive impact of economic improvement and meeting human needs, industrial development has raised concerns about clean water availability due to the discharge of wastewater into the environment [1,2]. One of the most dangerous categories of pollutants in industrial wastewater is the category of heavy metals, which are highly toxic and resistant to natural degradation. The existence of heavy metals in water is due to the fact that wastewater from various industries (e.g., mining, smelting, fuel or energy production, and others) is discharged into the environment. The presence of heavy metals in water can result in serious health consequences for both humans and other living organisms. Therefore, it is necessary to treat wastewater that contains heavy metal ions before discharging it into the environment [2,3]. Industries can produce wastewater with significant amounts of heavy metals, thus necessitating a strong focus on treating heavy metal-polluted water and wastewater from the outset [4].

Many treatment methods have been studied and applied for the removal of metal ions from wastewater, such as coagulation/flocculation, ion exchange, photocatalysis, flotation, electro-remediation, and others [5,6]. All treatment methods have both advantages and disadvantages and are presented in Table 1 [7]. However, adsorption is much preferred by researchers due to its high removal efficiencies, easy operation, and relatively low energy consumption [8]. The use of biomass sources as adsorbent materials for wastewater treatment supports circular economy systems and environmental sustainability [4,9,10]. Algae, such as *Sargassum* sp./*Padina* sp., *Caulerpa scalpelliformis, Pterocladia capillacea, Spirulina platensis,* and *Ulva lactuca,* were studied using simulated wastewater as well as raw wastewater and presented high water treatment efficiencies by removing heavy metal ions [11]. The advantages of using biomass in the process of removing metal ions from wastewater are presented through good reusability, high metal uptake, cost-effectiveness, and wide availability in nature [12,13].

Wastewater containing various heavy metals from different industries such as surface finishing, metal mining and smelting, energy production, fuel production, metallurgy, fertilizer and pesticide application, steelmaking, electrolysis, leather processing, electro-osmosis, production of electrical appliances, photography, metal surface treatment, and others are discharged into the environment [14]. Table 2 summarizes some heavy metals found in wastewater, focusing on their major sources, health effects, and the amount allowed in wastewater.

Lead (Pb) is a heavy metal commonly present in wastewater generated by industries such as battery manufacturing, piping, glass production, and ceramics. Lead can exist in two oxidation states, namely Pb (IV) and Pb (II), both of which can have harmful effects on human health by damaging the brain, circulatory system, and nervous system [15]. Additionally, lead can accumulate in soils for extended periods, lasting hundreds of years and negatively affecting food chains in nature and plant photosynthesis [16]. NTPA 001/2002 has set the maximum allowable concentration of lead in wastewater at 0.20 mg/L [17].

The main sources of manganese ions from wastewater are production of batteries, iron and steel alloys, glass production, and others [18]. Manganese ions can have harmful effects on human health by damaging the brain or respiratory tract. The NTPA 001/2002 standard has set the permitted concentration of Mn^2+^ in wastewater to 1.00 mg/L [17].

Conventional methods such as filtration, chemical precipitation, ion exchange, membrane technologies, electrochemical treatment, evaporation, adsorption on activated carbon, etc., have been used to remove metal ions from wastewater. However, if the concentrations of metal ions in wastewater are below 100 mg/L, electrochemical treatment and chemical precipitation are ineffective. Another disadvantage is that these processes result in a large amount of sludge which is difficult to treat. Activated carbon adsorption, membrane technologies, and ion exchange are very expensive processes and cannot be applied on a large scale [19].

Particular attention has been paid in recent years to the application of biotechnology for the removal of heavy metal ions from wastewater. Biosorption is an alternative process using certain natural materials that have a biological origin, including fungi, bacteria, algae, yeasts, etc. Biosorbents have metal ion trapping properties and can be used even when the concentrations of metal ions present in wastewater are very low. The efficiency of biosorbents is high, and they are ideal for treating large volumes of water with low concentrations of metal ions [14].

There are numerous studies investigating biosorbents that are used for the removal of organic substances and metal ions from wastewater. Biosorbents can be classified as fungi (for example, *Rhizopus arrhizus*), bacteria (for example, *Bacillus subtillis*), algae (for example, *Sargassum fusiforme* and *Enteromorpha prolifera*), yeast (for example, *Saccharomyces cerevisiae*), agricultural wastes (for example, corn cob), industrial wastes (for example, *Saccharomyces cerevisiae* waste biomass from the food industry and fermentation), and other polysaccharide materials [20].

**Table 2:** Information about typical heavy metals in wastewater and the loading limit values recommended by NTPA 001/2002.

Heavy Metals	Main Sources [18]	Main Organ and System Affected [18]	Permitted Quantities [mg/dm^3^] [17]
Lead (Pb^2+^)	Alloys, solder, lead-based batteries, ammunition, rust inhibitors, cable coating pigments, glazes, and plastic stabilizers.	Liver, bones, brain, kidneys, spleen, lungs, hematological system, immunological system, reproductive system, and cardiovascular system.	0.20
Manganese (Mn^2+^)	Batteries, iron and steel alloys, glass, various cleaning supplies, fireworks, fertilizers, fungicides, varnish, livestock feeding supplements, and cosmetics.	Brain and respiratory tract.	1.00
Copper (Cu^2+^)	Electronic and cables industry and corroded plumbing systems.	Brain, liver, cornea, kidneys, lungs, gastrointestinal system, hematological system, and immunological system.	0.10
Nickel (Ni^2+^)	Production of stainless steel and nickel alloys.	Kidneys, lungs, gastrointestinal system, and skin.	0.50
Arsenic (As^+^)	Glass and electronics production.	Lungs, skin, kidneys, brain, cardiovascular system, metabolic system, endocrine system, and immunological system.	0.10
Zinc (Zn^2+^)	Rubber products, brass coating, and some cosmetics.	Stomach and skin.	0.50
Cadmium (Cd^2+^)	Paints, batteries, corroded galvanized pipes, plastics industry, steel industry, and metal refineries.	Liver, bones, lungs, kidneys, brain, cardiovascular system, and immunological system.	0.20
Chromium (Cr) Cr^3+^/Cr^3+^+Cr^6+^/Cr^6+^	Steelworks, pulp mills, and tanneries.	Lungs, skin, liver, kidneys, pancreas, brain, taste, reproductive system, and gastrointestinal system.	-/1.00/0.10
Mercury (Hg^2+^)	Electrolytic production of caustic soda and chlorine, electrical appliances, refineries, laboratory apparatus, industrial and control instruments.	Lungs, brain, liver, kidneys, cardiovascular system, immunological system, and reproductive system.	0.05

The term phytoremediation comes from the Greek prefix “phyto”, meaning plants, and the Latin suffix “remedium”, meaning clean [21]. It is an in situ method that utilizes the ability of plants to absorb metals and organic pollutants from water, soil, and air [22]. Phytoremediation involves selecting the roots of plants that can access nutrients and metal ions through absorption, which can then be sequestered, immobilized, mobilized, or degraded. This remediation technique directly utilizes solar energy, eliminating the need for expensive equipment [23]. Phytoremediation plants share common properties such as fast growth involving high biomass production, extensive root systems, high tolerance, the ability to accumulate pollutants, and adaptability, and they are classified into three categories: indicators (for poor metal uptake and transport), excluders (for metal-sensitive plants), and accumulators (for higher uptake and accumulation). Plants belonging to the excluder groups are sensitive to heavy metals in a wide range of concentrations. They survive through restriction mechanisms [24]. Hyperaccumulators are plants that can tolerate elevated heavy metal concentrations and accumulate damaging heavy metals in their tissues [25].

It was reported that approximately 400 plant species from 45 different families have been identified as hyperaccumulators of metals, with Fabaceae, Asteraceae, Brassicaceae, Scrophulariaceae, Euphorbiaceae, and Lamiaceae being the most common families [26]. Several plants were identified with high bioaccumulation potential for cadmium/zinc, cobalt, nickel, and selenium including *Thlaspi caerulescens* (*Brassicaceae*) [27,28], *Haumaniastrum robertii* (*Lamiaceae*) [27,29], *Sebertia acuminata (Sapotaceae)* [27,30] and *Astragalus racemosus (Fabaceae)* [27,31]. Plants such as *Salix (Salicaceae)* [32], *Corn* (*Zea mays*) [33], and *Brassica juncea (Brassicaceae*) [34] have been found to exhibit elevated heavy metal tolerance and uptake efficiency. Many plants accumulate heavy metals in both their vegetative and reproductive parts, with some, such as *Brassica oleracea L., Nicotiana tabacum L.,* and *Lactuca sativa L*. accumulating high levels of cadmium in their leaves rather than roots. *Brassica juncea (Brassicaceae)* [34] roots can effectively eliminate cadmium, copper, chromium, lead, nickel, and zinc, while *Helianthus annuus (Asteraceae)* [35] can remove lead, cesium, uranium, and strontium from hydroponic solutions [36,37].

Phytoremediation has several benefits, including its autotrophic nature and ability to produce large biomass, which requires minimal nutrient input and is simple to manage. Additionally, the use of plants for remediation purposes is widely accepted by society due to the environmental sustainability and their aesthetic appeal [38].

Typically, the aboveground parts of plants are responsible for the accumulation, concentration, and translocation process. The process begins with the extraction of metals from the soil solution, which is then mobilized towards the root surface. Heavy metal ions are selectively bound to root cells and transported to aboveground parts of the plant, facilitating metal uptake through the roots. The transport process is facilitated by transporter proteins and occurs within the vascular system of the plant [38,39]. In aquatic ecosystems, phytoremediation can occur through direct absorption from wastewater, in function of the type of hyperaccumulator plant and of the level of pollution. When aquatic plants come into direct contact with polluted wastewater, passive heavy metal ions transport processes are initiated, resulting in heavy metal ion accumulation in the aboveground parts of the plant [40].

In phytoremediation, the process of heavy metal ion uptake and transport in plants can be divided into four types: phytostabilization, phytofiltration, phytoextraction, and phytovolatilization. Phytostabilization reduces the mobilization of metals; phytofiltration involves adsorption and/or absorption of metal ions by plant parts from the aqueous environment; phytoextraction involves heavy metal ions uptake from the environment into the biomass of the plant; and phytovolatilization involves the conversion of some heavy metals into less dangerous elements within the plant and their release into the atmosphere through leaves [41]. The phytoremediation process can also reduce pollution through mechanisms, namely, alteration in root exudates, increased biodegradation with root tissues, antimicrobial degradation, and abiotic losses [42]. The rhizosphere and the microbes in the roots can enhance the phytoremediation process by improving metal translocation and reducing the mobilization of metals [43]. However, the efficiency of phytoremediation is often limited by factors such as low biomass production at higher heavy metal ion concentrations, slow growth rate, sensitivity toward multi-metals, and poor and shallow root systems. Chemical reagents and agricultural approaches have been used to increase biomass and stimulate heavy metal ion uptake in plants, ultimately enhancing remediation efficiency [44].

Marine algae comprise about 250 genera and 1500 species. The second largest species in the seaweed category are brown algae [45,46]. *Sargassum fusiforme* belongs to the order *Fucales*, family *Sargassaceae*. It is an edible brown seaweed that can be found in the temperate Pacific Northwest. *Sargassum fusiforme* is distributed along the coasts of Korea, Japan, and China [47,48]. This seaweed contains various complex nutrients and therefore has been used as a therapeutic drug and essential food for thousands of years [49]. To see if the algae are free of adsorbed heavy metals, they can be tested using various methods, simpler or more complex, such as optical imaging spectroscopy [50] or dry ashing method [51].

*Enteromorpha* is a green seaweed that belongs to the phylum *Chlorophyta*, the class *Chlorophyceae*, and the order *Ulvales* [52]. *Enteromorpha* genus comprises different species of green algae namely *E. prolifera, E. linza, E. intestinalis, E. compressa*, and *E. flexuosa*. *E. prolifera* has been shown to be the dominant species in the Yellow Sea of China [53]. It has been used as a functional food and a traditional medicine [54,55,56,57,58].

Table 3 shows the results reported by researchers from experimental analyses using different types of algae.

Compared to the experimental results reported by other scientists, the treatment efficiencies presented in the experimental research part of this article are higher; even the complete removal of lead ions from wastewater was achieved. It can be seen that the treatment time required in our experimental research was higher than the treatment time reported by other scientific researchers. The temperature at which the experiments were carried out was room temperature, the same as the temperature reported by most scientific researchers in their work. It has been observed that algae are very effective for removing metal ions from wastewater in an acidic pH environment.

Algae can be used as a raw material for different industrial purposes, such as biofuel production. Thermochemical processes such as gasification, fast pyrolysis, lipid transesterification, and [68] hydrothermal liquefaction (HTL) achieve the conversion of algae to biofuels [69].

The novelty in the experimental research is represented by the study of manganese ion removal from wastewater using *Sargassum fusiforme* and *Enteromorpha prolifera*. In addition, the concentrations of lead and manganese ions studied are different from those presented by other researchers who have removed metal ions from wastewater using algae.

## 2. Materials and Methods

**Figure 1:**
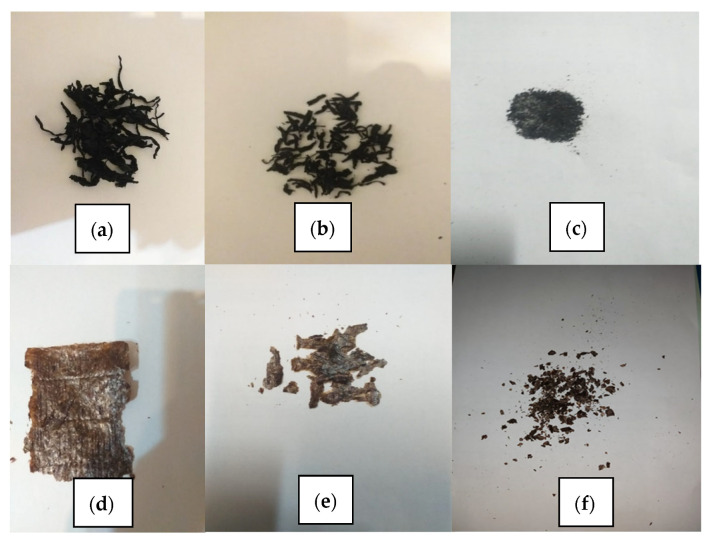
(**a**) Sargassum filiforme (native form); (**b**) Sargassum filiforme (coarse form); (**c**) Sargassum filiforme (ground form); (**d**) Enteromorpha (Ulva) prolifera (native form); (**e**) Enteromorpha (Ulva) prolifera (coarse form); (**f**) Enteromorpha (Ulva) prolifera (ground form).

The algae biosorbent was used in three forms, namely native, coarse, and ground (Figure 1) for the removal of metal ions from wastewater. These three forms were used because the specific surface area of algae changes when their form changes. Thus, if the specific surface area is larger, the treatment efficiencies will be higher. The native form of algae refers to the natural state, the coarse form involves cutting the algae in its native form to a size of 2 cm, and the ground form involves grinding the native form in a mortar using a pestle. Standard solutions of manganese and lead of 1000 mg/L were used for the preparation of wastewater. Continuous agitation of the water was carried out using a mechanical stirrer.

To remove lead and manganese ions from wastewater, 1 g of algae was used. The initial concentration of manganese ions was 1.64 mg/L, and the initial concentration of lead ions studied was 1.86 mg/L. Concentrations were obtained by performing dilutions using standard solutions of manganese and lead. The phytoremediation process was carried out under continuous stirring, at room temperature. Samples were taken every 2 h and prepared for concentration determination using the PhotoLab S12 photometer, in 10 mm quartz cuvette.

In order to prepare the sample for the determination of lead ion concentrations, 0.50 mL of solution containing potassium cyanide and 0.50 mL of solution containing hydroxylammonium chloride and ammonia solution were added to a test tube, to which 8 mL of sample was added. 

For the determination of manganese ion concentrations at the photometer, the pH of the wastewater studied must be between 2 and 7. Thus, the pH of the samples taken is checked and adjusted if necessary. After this operation, 5 mL of sample is pipetted into a test tube over which a solution containing formaldehyde and hydroxylammonium chloride is added in drops. After sample homogenization, it is allowed to react for 2 min. The sample is transferred from the test tube into a 10 mm quartz cuvette, positioned in its dedicated place inside the photometer, and the concentration of manganese ions is determined.

Taking into consideration the determined concentrations, the treatment efficiencies were calculated using the following formula:

(1) $$\eta =\frac{C_{i}-C_{f}}{C_{i}}*100$$

where η–represents the wastewater treatment yield, %; *C_i_*-represents the initial concentration of lead or manganese ions, mg/L; *C_f_*-represents the final concentration of lead or manganese ions, mg/L.

## 3. Results and Discussion

**Figure 2:**
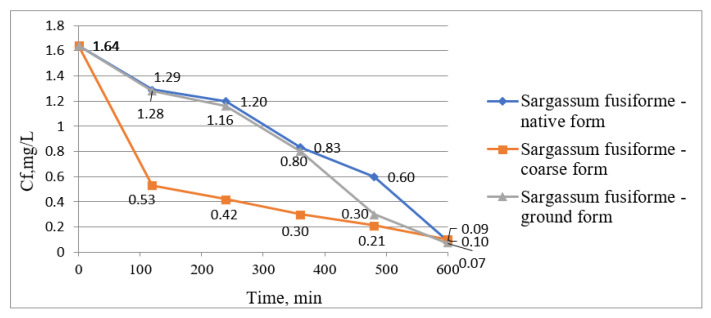
Mn (II) ion concentration variation in wastewater over time using *Sargassum fusiforme*.

**Figure 3:**
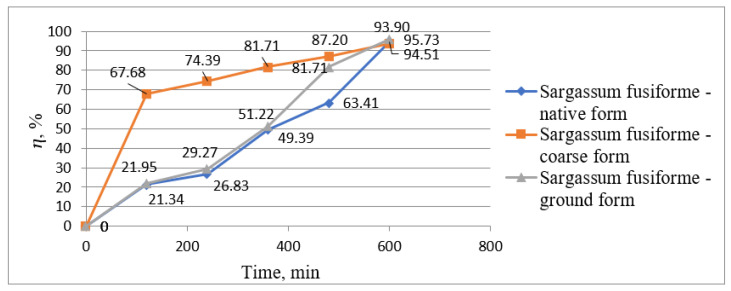
The removal efficiencies of Mn (II) ions from wastewater variation over time using *Sargassum fusiforme*.

Figure 2 and Figure 3 show graphically the results obtained from the experimental research on the removal of manganese ions from wastewater using *Sargassum fusiforme* in three forms, namely native form, coarse form, and ground form. Figure 2 shows graphically the values of manganese ion concentrations determined from samples taken every 2 h, and Figure 3 presents graphically the treatment efficiencies calculated using the yield formula according to Equation (1).

Figure 2 presents the decrease in manganese ion concentration in wastewater from the initial concentration of 1.64 mg/L to the final concentrations of 0.09, 0.1, and 0.07 when *Sargassum fusiforme* was used in native, coarse, and ground form, respectively. Concentrations decreased gradually over a period of 600 min contact time.

The increase in treatment efficiency through the phytoremediation process is presented in Figure 3. *Sargassum fusiforme* in its coarse form was very efficient in the first period, reaching 67.68% in the first 120 min of contact time, while the other two forms of *Sargassum fusiforme* showed efficiencies of only 21.34 and 21.95% in this period. However, at the end of the phytoremediation process, the highest treatment efficiency was obtained using *Sargassum fusiforme* in the ground form, i.e., 95.73%. In addition, at the end of the process, in all three cases, the yields were similar, i.e., 93.90, 94.51, and 95.73% using *Sargassum fusiforme* in coarse, native, and ground form, respectively.

**Figure 4:**
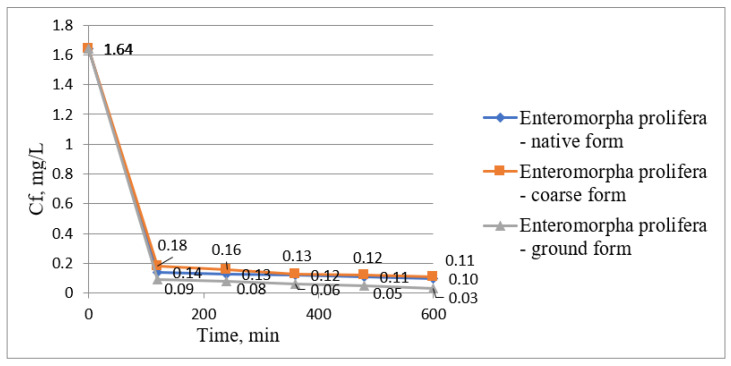
Variation graph of Mn (II) ion concentration over time using *Enteromorpha prolifera*.

**Figure 5:**
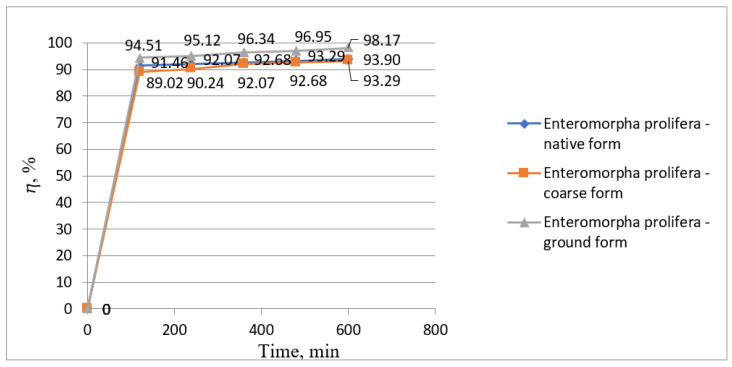
Variation chart of the removal efficiencies of Mn (II) ions from wastewater over time using *Enteromorpha prolifera*.

In Figure 4, the concentrations of manganese ions determined during the whole phytoremediation process were plotted; in Figure 5, the treatment efficiencies using *Enteromorpha prolifera* in three forms, namely, native form, coarse form, and ground form, were presented.

Using *Enteromorpha prolifera*, in all forms presented above, showed a fast removal of manganese ions from wastewater within the first 120 min of contact time, reaching manganese ion concentrations of 0.14, 0.18, and 0.09 mg/L from an initial concentration of 1.64 mg/L Mn^2+^. By the end of the phytoremediation process, the manganese ion concentrations in the water gradually decreased to concentrations of 0.10, 0.11, and 0.03 mg/L, respectively, using *Enteromorpha prolifera* in native form, coarse form, and ground form (Figure 4).

It can be seen from Figure 5 that treatment yields quickly reached 91.46, 89.02, and 94.51%, respectively, in the first 120 min of contact time using *Enteromorpha prolifera* in native form, coarse form, and ground form. The treatment efficiencies gradually reached 93.90, 93.29, and 98.17%, respectively, in 600 min of contact time.

**Figure 6:**
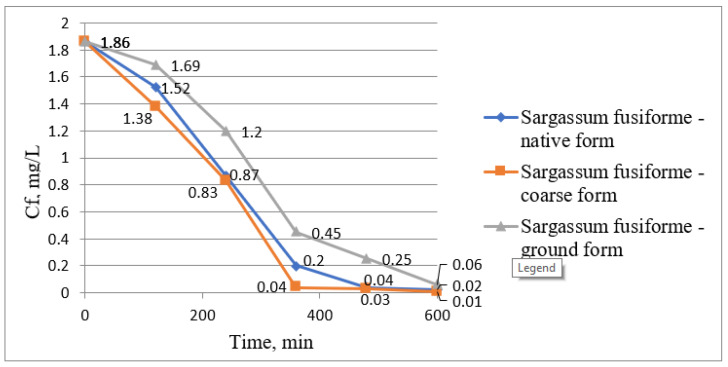
Pb (II) ion concentrations variation graph over time using *Sargassum fusiforme*.

**Figure 7:**
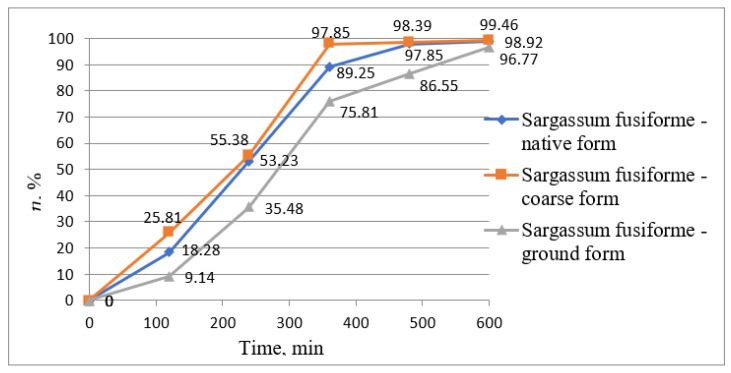
Pb (II) ion removal efficiency variation graph from wastewater over time *using Sargassum fusiforme*.

**Figure 8:**
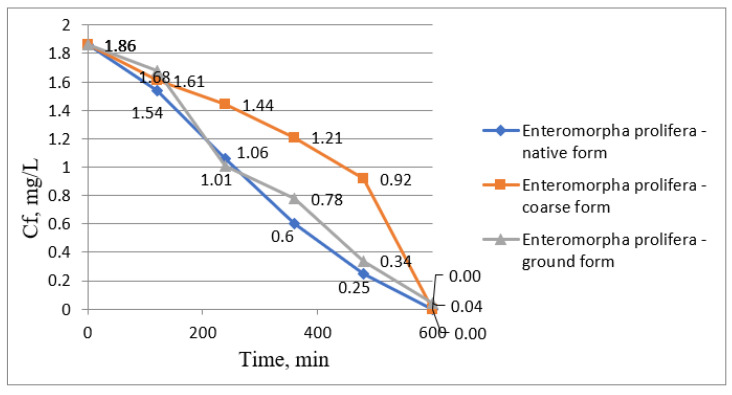
Pb (II) ion concentration variation graph over time using *Enteromorpha prolifera*.

**Figure 9:**
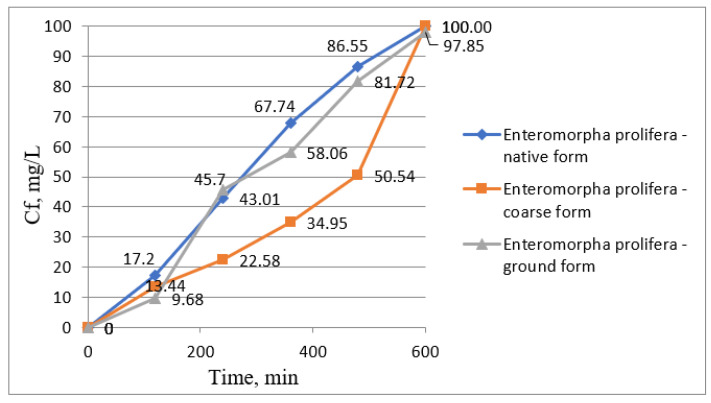
Pb (II) ion removal efficiency variation graph from wastewater over time using *Enteromorpha prolifera*.

The removal of lead ions from wastewater was also studied using the same algae presented above, namely *Sargassum fusiforme* and *Enteromorpha prolifera*. The results obtained are presented graphically in the following figures. Figure 6 represents the variation of lead ion concentrations over time using *Sargassum fusiforme*, Figure 7 represents the variation of removal yields over time using the same algae, Figure 8 represents the variation of lead ion concentrations over time using *Enteromorpha prolifera*, and Figure 9 represents the variation of removal yields over time using also *Enteromorpha prolifera*.

Figure 6 shows the gradual decrease in lead ion concentrations in wastewater in 600 min from the initial concentration of 1.84 mg/L to the final concentrations of 0.02, 0.01, and 0.06 when using *Sargassum fusiforme* in native, coarse, and ground form, respectively.

The increase in treatment efficiency through the phytoremediation process for the removal of lead ions from wastewater is shown in Figure 7. At the end of the treatment process, the treatment efficiency that was close to the maximum efficiency was obtained using *Sargassum fusiforme* in coarse form, namely 99.46%. However, in all three cases, the treatment efficiencies were similar, namely 99.46, 98.92, and 96.77% using *Sargassum fusiforme* in coarse, native, and ground form, respectively.

Lead ion concentrations gradually decreased until lead ions were completely removed from wastewater when *Enteromorpha prolifera* was used in its native and coarse form. The final concentration of lead ions in the waters when *Enteromorpha prolifera* was used in ground form was 0.04 mg/L (Figure 8).

It can be seen from Figure 9 that maximum treatment yields were obtained using *Enteromorpha prolifera* in native and coarse forms in a period of 600 min contact time.

**Figure 10:**
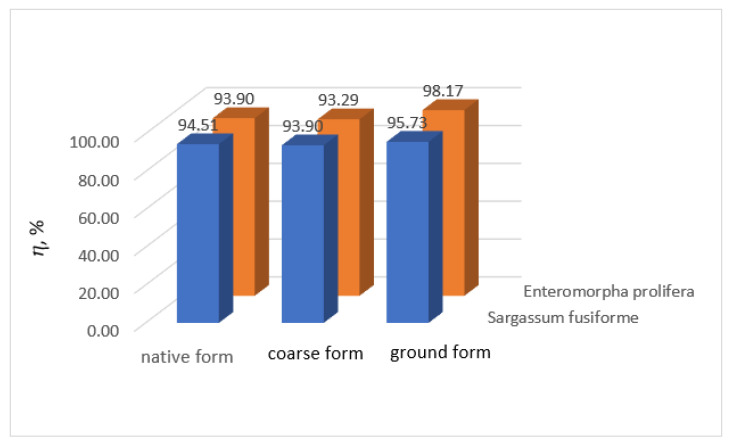
Comparison of manganese ion wastewater treatment efficiencies using *Sargassum fusiforme* and *Enteromorpha prolifera*.

**Figure 11:**
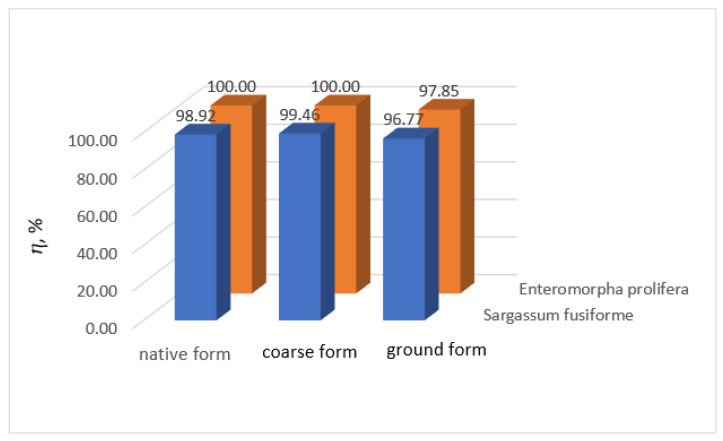
Comparison of lead ion water treatment efficiencies using *Sargassum fusiforme* and *Enteromorpha prolifera*.

Comparison of the wastewater treatment percentage obtained using the two algae, in their three studied forms, for the removal of lead and manganese ions from wastewater are plotted in Figure 10 and Figure 11, respectively.

Comparing the wastewater treatment efficiencies obtained for the removal of manganese ions from wastewater using *Sargassum fusiforme* and *Enteromorpha prolifera*, we observed that the results are quite similar. Using *Enteromorpha prolifera*, the wastewater treatment efficiencies vary from 93.29% to 98.17%, and using *Sargassum fusiforme,* the wastewater treatment efficiencies vary from 93.90% to 95.73%.

In the case of lead ion removal from wastewater, comparing the results, the treatment efficiencies were higher in all three cases when using *Enteromorpha prolifera* than when using *Sargassum fusiforme*. The cell walls of algae are made of various substances, which may influence the biosorption efficiency [70,71]. The cell walls of brown algae (e.g., *Sargassum fusiforme*) generally contain three components: guluronic acids, alginic acids, and cellulose. The walls of green algae (e.g., *Enteromorpha prolifera*) are mainly composed of cellulose [70,72]. This may explain why efficiencies of *Enteromorpha prolifera* were higher and *Sargassum fusiforme* were lower.

The results obtained from the removal of lead and manganese ions from wastewater are closely related to the metabolic bioaccumulation mechanism of algae. The living cells of algae control the internalization of metal. The metal homeostasis system controls the concentration of metal that has been internalized. Within a short period of time, algal cells try to sequester an unwanted metal in vacuoles or in other intracellular organelles for possible export from the cell using efflux systems. Three mechanisms are suitable for the biosorption process: adsorption, ion exchange, or microprecipitation. It has been shown that dead algal cells are much more efficient at removing heavy metal ions from wastewater than live algal cells [73].

## 4. Conclusions

Water pollution with heavy metal ions from various industries is increasing and is a real problem for the environment. In conclusion, the use of plants and in particular algae to treat water containing heavy metal ions is very effective. Phytoremediation is an increasingly used method because it is inexpensive and has very high treatment efficiencies.

The use of *Sargassum fusiforme* and *Enteromorpha prolifera* has proven to be an excellent option for the removal of lead and manganese ions from wastewater. The wastewater treatment efficiency was 100% when *Enteromorpha prolifera* was used for the removal of lead ions from wastewater, and the treatment time necessary for this experiment was only 10 h. Using this alga, 98.17% of manganese ions were removed from wastewater. *Sargassum fusiforme* was also effective, removing 95.73% of manganese ions and 99.46% of lead ions from wastewater. The most important aspect to keep in mind is how the algae are used, whether in their native, coarse, or ground form. However, the differences in treatment efficiencies were not major. Compared to experimental results reported by other researchers, the treatment time that was presented in the experimental part of this article was longer, but the efficiencies for the removal of manganese and lead ions from wastewater were higher.

## Figures and Tables

**Table 1:** Advantages and disadvantages of some water treatment methods.

Water Treatment Methods	Advantages	Disadvantages
Coagulation/ flocculation	Sludge settling, process simplicity	Generation of large volume of sludge, high operational costs
Ion exchange	Simple equipment, easy control and maintenance	High operational costs
Photocatalysis	No sludge production, less harmful byproduct, instant removal of metals and organic pollutants	Limited application
Flotation	High efficiency for removing pollutants, rapid operation, inexpensive	High cost of operation and maintenance
Electrochemical treatment	Efficient technology for the recycling/recovery of valuable metals	High capital and operating costs

**Table 3:** Types of algae used to remove metal ions from wastewater and experimental conditions.

Algae	Heavy Metals	Time [min]	pH	Temperature [°C]	Yield [%]	Ref.
*Sargassum crassifolium*	Cd (II), Hg (II), Pb (II)	60	2, 3, 4, 5, 9	-	75.00–99.05	[59]
*Sargassum myriocystum*	Pb (II)	60	5	25	89.75	[60]
*Sargassum* sp./*Padina* sp.	Pb (II), Cu (II)	~60	5	-	90.00	[61]
	Cd (II), Zn (II), Ni (II)		5.5			
Formaldehyde-treated *Cystoseira indica biomass*	Cd (II), Ni (II)	~180	6	25	90.00	[62]
Formaldehyde-treated *Nizimuddinia zanardini biomass*	Pb (II)		5.5			
Formaldehyde-treated *2-Hypnea valintiae*	Co (II)	120	6	25	~90.00	[63]
*Caulerpa scalpelliformis*	Zn (II)	-	5.7	30	89.60	[64]
*Chlorella minutissima*	Zn (II)	20	6	28	62.00	[65]
*Pterocladia capillacea*	Cr (III)	45	1	25	80.00–85.00	[66]
*Spirulina platensis*	Cu (II)	90	7	37	90.60	[67]
*Scenedesmus quadricauda*	Cr (VI)	-	1	-	60.00	
*Scenedesmus quadricauda*	Cr (III)	-	6	-	85.00	
*Dunaliela*	Cd (II), Pb (II), Ni (II), Cr (II), Zn (II), Cu (II)	-	-	-	74.00–95.00	
*Jania rubens*	Hg (II)	60	6	-	54.00–71.00	
*Ulva lactuca*	Cr (VI)	15–180	5	-	96.00	
*Ulva lactuca*	Hg (II)	60	6	-	60.00–86.00	
*Sphaerococcus* *coronopifolius*	Hg (II)	60	6	-	70.00–90.00	
*Azolla fliculoides*	Cr (VI)	100	2	-	83.00	
*Caulerpa fastigiata*	Pb (II)	90	5	-	70.00–82.00	
*Sargassum myriocystum*	Pb (II)	60	5	25	87.00	
*Osmundea pinnatifda*	Cu (II)	60	5	-	70.00	
*Osmundea pinnatifda*	Cd (II)	60	5	-	75.00	
*Cystoseira indica*	Co (II), Cu (II)	70	-	45	90.00	

## Data Availability

Not applicable.
